# Editorial: Rare hematological malignancies

**DOI:** 10.3389/fcell.2025.1600063

**Published:** 2025-04-23

**Authors:** Pier Paolo Piccaluga

**Affiliations:** ^1^ Biobank of Research, IRCCS Azienda Ospedaliera-Universitaria di Bologna Policlinico di S. Orsola, Bologna, Italy; ^2^ Department of Medical and Surgical Sciences, Bologna University School of Medicine, Bologna, Italy

**Keywords:** rare hematological malignancy, peripehral T-cell lymphomas, multiple myeloma, hematopoietic stem cell transplantation, novel agents, maintenance therapy, chronic myeloproliferative neplasm, Large granular lymphocytic (LGL) leukemia

Hematological malignancies present a diverse group of cancers with varying prognoses and treatment responses (Alaggio et al., 2022; Khoury et al., 2022). While significant strides have been made in understanding and treating common hematological malignancies, rare subtypes continue to pose significant clinical challenges. This Research Topic of Frontiers in Cell and Developmental Biology delves into the intricacies of these rare hematological malignancies, exploring their pathogenesis, prognostic factors, and potential therapeutic avenues. From investigating the role of HDAC10 in Sézary Syndrome to understanding the implications of CALR mutations in chronic myeloproliferative neoplasms, the articles presented here highlight the importance of dedicated research in advancing our knowledge and improving patient outcomes in these rare diseases. Furthermore, this Research Topic underscores the value of international collaborations, as exemplified by the study on primary lung extranodal NK/T-cell lymphoma utilizing data from both China and the SEER database. By shedding light on these rare subtypes, this Research Topic aims to encourage further research and ultimately contribute to the development of more effective and targeted therapies for patients battling these challenging diseases.

One of the most relevant Research Topic, when dealing with scientific advancements in rare cancer, is the common lack of suitable pre-clinical models, as in the case of T-cell lymphomas (TCL). TCL represent about 10% of all human lymphomas, being relatively rarer in Western Countries than in Asia. Remarkably, they are quite heterogeneous, including roughly 30 different entities characterized by different presentation (nodal vs. leukemic vs. extra nodal) and clinical aggressiveness (from indolent to quickly fatal) (Piccaluga and Khattab, 2023). In their article, Luo et al. provided a comprehensive review of the evolving landscape of *in vivo* mouse models employed to elucidate the pathogenesis of cutaneous T-cell lymphoma (CTCL), a complex and heterogeneous hematological malignancy (1372881). By systematically categorizing and evaluating various model systems, including genetically engineered mouse models (GEMMs), xenografts, and syngeneic transplantation approaches, the authors underscore the critical role these models play in advancing our understanding of CTCL’s intricate tumor-host interactions and therapeutic responses. Notably, the discussion highlights the limitations of current models, particularly their challenges in fully recapitulating human immune responses and the early stages of disease progression. The introduction of skin-targeted GEMMs represents a significant advancement, allowing for a more accurate depiction of the disease’s initiation and development within the skin microenvironment. This work not only emphasizes the necessity for a balanced assessment of the strengths and weaknesses of existing models but also illustrates the potential of innovative approaches to accelerate the identification of CTCL-related genetic factors and the development of targeted therapeutic strategies, thus addressing a pressing need in the field of oncology.

Another rare T-cell malignancy was the object of another contribution (1434676). In their article, Gorodetskiy et al. presented a comprehensive retrospective analysis of gamma-delta T-cell large granular lymphocytic leukemia (γδT-LGL leukemia) (Piccaluga and Khattab, 2023) in patients with rheumatologic diseases, particularly rheumatoid arthritis (RA), elucidating both clinical and genetic characteristics that underscore the intricate relationship between these conditions. The findings reveal that a significant proportion of patients exhibited manifestations of γδT-LGL leukemia either prior to or concurrently with the onset of RA, suggesting a potential contributory role of the leukemia in the pathogenesis of the autoimmune condition. Moreover, the high incidence of STAT3 mutations in this cohort (detected in 80% of patients) aligns with existing literature that correlates these genetic alterations with the development of RA, thereby reinforcing the notion of a shared pathogenic pathway. The study also highlights the diagnostic challenges posed by atypical presentations of γδT-LGL leukemia, particularly in cases characterized by splenomegaly and neutropenia without detectable tumor cells in peripheral blood, which could lead to misdiagnosis as other hematological malignancies. By providing critical insights into the immunophenotypic profiles and molecular landscape of this rare leukemia, the research not only enhances our understanding of its clinical manifestations but also raises important considerations for future investigations into the causal mechanisms linking γδT-LGL leukemia and autoimmune diseases, ultimately contributing to more tailored therapeutic approaches in affected patients.

The submission by Pieniawska et al. then introduced significant findings that elucidate the role of HDAC10 as a pivotal epigenetic regulator in the context of Sézary Syndrome, a rare and aggressive form of cutaneous T-cell lymphoma characterized by the infiltration of malignant T-cells into the skin and blood (Piccaluga and Khattab, 2023) (1480192). The study reveals that HDAC10 is overexpressed in Sézary Syndrome patients, predominantly localized in the cytoplasm, where it appears to inhibit apoptosis and modulate critical cellular processes such as autophagy and cell proliferation. Importantly, the research demonstrates that knockdown of HDAC10 not only reduces cell growth but also triggers apoptosis, underscoring its potential as a therapeutic target. Furthermore, the whole transcriptome analysis correlates HDAC10 expression with key cancer-related signaling pathways, including the PI3K-Akt and JAK-STAT pathways, which are crucial for malignant T-cell survival. The findings suggest that selective inhibition of HDAC10 may enhance the sensitivity of Sézary cells to pro-apoptotic agents like Camptothecin, highlighting a promising avenue for combinatorial therapeutic strategies. This work not only contributes to a deeper understanding of the molecular mechanisms underlying Sézary Syndrome but also opens new horizons for the development of targeted therapies aimed at improving treatment outcomes in this challenging malignancy.

Remaining in the same field of T-cell lymphomas, Li et al. performed a retrospective analysis of data from China and the SEER database to assess prognostic factors for primary lung extranodal NK/T-Cell lymphomas, providing pivotal insights into such an extremely rare and aggressive form of non-Hodgkin lymphoma (1496735). The study elucidates the complex interplay between clinical characteristics and survival outcomes, identifying sex as a significant prognostic factor, thereby challenging conventional paradigms that often prioritize disease stage and age. Additionally, the research integrates data from a diverse patient cohort, enhancing the generalizability of its findings and underscoring the critical need for further investigations into the molecular and immunological underpinnings of this malignancy. By highlighting both the diagnostic challenges and the urgent need for tailored therapeutic strategies, this work not only advances our understanding of primary lung ENKTL but also sets the stage for future research to unravel the intricate biological mechanisms that drive this rare lymphoma, ultimately aiming to improve clinical outcomes and therapeutic interventions.

The review article by Zhang et al. presented a comprehensive analysis of the pivotal role of natural killer (NK) cells in the pathogenesis and treatment of multiple myeloma (MM) (1359084), a malignancy characterized by the clonal proliferation of plasma cells and a significant interaction between the clone and the surrounding microenvironment. Indeed, MM is probably the first example of cancer in which treating the microenvironment significantly modified the natural history of the disease in the past 10–15 years. In their article the authors elucidate how NK cells, with their germline-encoded receptors, contribute to the immune surveillance of aberrant plasma cells during the early stages of malignant transformation from monoclonal gammopathy of undetermined significance (MGUS) and smoldering multiple myeloma (SMM) to active disease. Moreover, the review highlights the dynamic interplay between NK cells and various therapeutic modalities, including immunomodulatory drugs, proteasome inhibitors, and monoclonal antibodies, emphasizing the dual role of NK cells as both effectors in anti-tumor responses and as modulators of the tumor microenvironment that may contribute to drug resistance and disease relapse. This comprehensive examination underscores the necessity for further investigation into NK cell dynamics and their potential therapeutic applications, advocating for strategies that harness NK cell function to overcome the challenges posed by drug-resistant MM. By integrating insights into NK cell biology with clinical implications, this review lays the groundwork for innovative combination therapies aimed at enhancing patient outcomes in MM.

Finally, the Research Topic included two case reports that, by highlighting the uique feature of selected cases, raised discussion on more general aspect of hematological cancers. Fang et al. illustrated a rare instance of massive splenomegaly resulting from primary myelofibrosis in a 72-year-old female patient, highlighting the intricate interplay and diagnostic challenges between hematological malignancies and the resultant abdominal organomegaly (1422776). The surgical intervention, characterized by an open splenectomy, underscores the critical role of multidisciplinary collaboration in managing complex cases where conventional therapies have failed. Notably, the application of Enhanced Recovery after Surgery (ERAS) protocols during the perioperative period demonstrated a significant advancement in optimizing patient outcomes through evidence-based practices aimed at minimizing surgical stress and facilitating recovery. The patient’s rapid postoperative recovery and resumption of daily activities not only emphasize the importance of tailored nursing care and nutritional optimization but also illuminate the potential for ERAS protocols to enhance recovery trajectories in surgical oncology settings. This report serves as a vital contribution to the understanding of splenic pathology in myeloproliferative disorders and underscores the necessity for ongoing research into integrative therapeutic strategies that can improve quality of life for patients afflicted by similar complex conditions.

Finally, an Italian group presented a comprehensive review of the rare coexistence of *BCR::ABL1* translocation and *CALR* mutations within myeloproliferative neoplasms (MPNs), elucidating the diagnostic and therapeutic implications of this unique genetic interplay (1391078). Despite the long-standing notion that *BCR::ABL1* and *CALR* mutations are mutually exclusive, this study highlights an emerging understanding of their concurrent presence, which has been documented in only 24 cases to date. By systematically analyzing clinical, pathological, and molecular features across these cases, the authors underscore the potential for misinterpretation of clinical manifestations, which may be mistakenly attributed to disease progression or resistance to targeted therapies. The interplay between the *BCR::ABL1* and *CALR*-mutant clones is particularly noteworthy, revealing contrasting responses to therapy that necessitate tailored management strategies. In addition, it underlined the complexity pf MPN genetics, as recently revealed by massive parallel sequencing studies (Visani et al., 2023). In this regard, this review not only enriches the existing literature but also calls for heightened awareness among clinicians and pathologists regarding this hybrid disease, advocating for comprehensive genetic screening and refined diagnostic criteria to optimize patient outcomes. The findings herein thus contribute significantly to our understanding of MPN pathogenesis and underscore the necessity for a nuanced approach in the therapeutic landscape of these complex disorders.

In conclusion, this Research Topic on Rare Hematological malignancies did provide some novel insights particularly as far as T-cell lymphomas are concerned. It did emerge that novel pre-clinical models are needed as well as large cooperative studies, relying on large databases in order to identify novel prognostic scores. Finally, the role of microenvironment as well as of complex genetic pattern in the pathogenesis of the diseases has been discussed promoting new ideas for future treatments.

## References

[B1] Alaggio R. Amador C. Anagnostopoulos I. Attygalle A. D. Araujo I. B. O. Berti E. (2022). The 5th edition of the world health organization classification of haematolymphoid tumours: lymphoid neoplasms. Leukemia 36 (7), 1720–1748. 10.1038/s41375-022-01620-2 35732829 PMC9214472

[B2] Khoury J. D. Solary E. Abla O. Akkari Y. Alaggio R. Apperley J. F. (2022). The 5th edition of the world health organization classification of haematolymphoid tumours: myeloid and histiocytic/dendritic neoplasms. Leukemia 36 (7), 1703–1719. 10.1038/s41375-022-01613-1 35732831 PMC9252913

[B3] Piccaluga P. P. Khattab S. S. (2023). A comparison of the fifth world health organization and the international consensus classifications of mature T-cell lymphomas. Int. J. Mol. Sci. 24 (18), 14170. 10.3390/ijms241814170 37762472 PMC10532420

[B4] Visani G. Etebari M. Fuligni F. Di Guardo A. Isidori A. Loscocco F. (2023). Use of next generation sequencing to define the origin of primary myelofibrosis. Cancers (Basel) 15 (6), 1785. 10.3390/cancers15061785 36980671 PMC10046249

